# RNASET2-deficient leukoencephalopathy mimicking congenital CMV infection and Aicardi-Goutieres syndrome: a case report with a novel pathogenic variant

**DOI:** 10.1186/s13023-019-1155-9

**Published:** 2019-07-26

**Authors:** Reyhaneh Kameli, Man Amanat, Zahra Rezaei, Sareh Hosseionpour, Sedigheh Nikbakht, Houman Alizadeh, Mahmoud Reza Ashrafi, Abdolmajid Omrani, Masoud Garshasbi, Ali Reza Tavasoli

**Affiliations:** 1 Myelin Disorders Clinic, Pediatric Neurology Division, Children’s Medical Center, Pediatrics Center of Excellence, Tehran University of Medical Sciences, Tehran, Iran; 2 Faculty of Medicine, Students’ Scientific Research Center, Tehran University of Medical Sciences, Tehran, Iran; 3 Department of Pediatrics, Division of Pediatric Radiology, Children’s Medical Center, Tehran University of Medical Sciences, Tehran, Iran; 4 Department of Medical Genetics, Faculty of Medical Sciences, Tarbiat Modares University, Tehran, Iran

**Keywords:** Ribonuclease, RNASET2-deficienct leukoencephalopathy, Cystic leukoencephalopathy, Aicardi-Goutieres syndrome, Congenital cytomegalovirus infection

## Abstract

**Background:**

Ribonucleases (RNases) are crucial for degradation of ribosomal RNA (rRNA). RNASET2 as a subtype of RNASEs is a 256 amino acid protein, encoded by *RNASET2* gene located on chromosome six. Defective *RNASET2* leads to RNASET2*-*deficient leukoencephalopathy, a rare autosomal recessive neurogenetic disorder with psychomotor delay as its main clinical symptom. The clinical findings can be similar to congenital cytomegalovirus (CMV) infection and Aicardi-Goutieres syndrome (AGS).

**Methods:**

Herein, we presented a patient with motor delay, neurological regression, infrequent seizures and microcephaly at 5 months of age. Brain imaging showed white matter involvement, calcification and anterior temporal cysts. Basic metabolic tests, serum and urine CMV polymerase chain reaction (PCR) were requested. According to clinical and imaging findings, screening of *RNASET2* and *RMND1* genes were performed. The clinical data and magnetic resonance imaging (MRI) findings of previous reported individuals with RNASET2-deficient leukodystrophy were also reviewed and compared to the findings of our patient.

**Results:**

Brain MRI findings were suggestive of RNASET2*-*deficient leukoencephalopathy, AGS and CMV infection. Basic metabolic tests were normal and CMV PCR was negative. Molecular study revealed a novel homozygous variant of c.233C > A; p.Ser78Ter in exon 4 of *RNASET2* gene compatible with the diagnosis of RNASET2*-*deficient leukoencephalopathy.

**Conclusions:**

RNASET2-deficiency is a possible diagnosis in an infant presented with a static leukoencephalopathy and white matter involvement without megalencephaly. Due to overlapping clinical and radiologic features of RNASET2-deficient leukoencephalopathy, AGS and congenital CMV infections, molecular study as an important and helpful diagnostic tool should be considered to avoid misdiagnosis.

## Background

Ribonucleases (RNases) are intracellular enzymes, catalyzing the degradation of ribosomal ribonucleic acid (rRNA). These enzymes are divided into different subtypes with multiple function [1–3]. RNASET2 as a defined subtype of RNases performs diverse roles in different species; including phosphate scavenging following nutritional stress and acting as cytotoxic agent. In human beings [2]. RNASET2 is localized within lysosomes and is a putative lysosomal hydrolase [4]. Mutation of *RNASET2* gene leads to the accumulation of undigested rRNA in lysosomes within the central nervous system which can cause a rare type of leukoencephalopathy [5].

RNASET2-deficient leukoencephalopathy also called ‘cystic leukoencephalopathy without megalencephaly’ is an autosomal recessive neurogenetic disorder which was first described in members of 5 families as a non-progressive neurological disease [6]. Most affected newborns were asymptomatic but psychomotor developmental delay with norm/microcephaly developed gradually during the first months of life. Lacking normal speech and intellectual disability were also observed in most cases. These individuals may show other neurological features including sensorineural hearing loss, seizures, spasticity, abnormal movements, and nystagmus [6–8].

Reported cases of RNASET2-deficient leukoencephalopathy showed clinical and neuroradiological features similar to congenital cytomegalovirus (CMV) infection and Aicardi-Goutieres Syndrome (AGS). Congenital CMV usually presents with seizures, microcephaly, and hydrocephaly. Brain imaging in these cases predominantly reveals multifocal white matter lesions, anterior temporal subcortical cysts, and intracranial calcifications [7]. AGS is an inherited disease associated with increased type 1 interferon activity in the serum and cerebrospinal fluid of patients [9]. Individuals with this type of rare diesease can also show encephalopathic features; including seizures, cognitive impairment and irritability in addition to microcephaly and altered muscle tone. Early onset forms of AGS can be associated with brain imaging features consisted of marked frontal / temporal lobe white matter involvement, anterior temporal lobe cysts, and intracranial calcification [8].

To date, twelve genetically confirmed cases of RNASET2-deficient leukoencephalopathy have been reported [6–8]. Herein, we described the clinical manifestations, brain imaging findings, as well as, genetic analysis results of another patient with RNASET2-deficient leukoencephalopathy due to a novel variant.

## Method

### Patient selection and ethical committee approval

We presented a genetically confirmed case of RNASET2-deficient leukoencephalopathy included in our database (Iranian Neurometabolic Disorders Registry, INMR); consisted of 285 individuals with different types of heritable white matter disorders. Informed consent was written by the parents of our participant. Ethical committee of Children’s Medical Center hospital approved our study.

### Participant

The participant is a 34 months old girl who was born to a consanguineous marriage at near term through an uneventful cesarean section with gestational age of 36 weeks with birth weight and head circumference of 2.7 Kg and 34 cm, respectively. According to the mother, patient’s sister died at 4 months of age due to a congenital heart disease but no medical documents were available. No medical condition was noticed in our participant until the age of 3.5 months. Tonic spasm seizures were the first manifestations of her disease and was under treatment with anti-seizure medications including phenobarbital, levetiracetam and diazepam. At 4 months of age, she visited our hospital (Children’s Medical Center) due to abnormal developmental milestones; particularly motor aspects and inability of neck holding. Her family was recommended to monitor their child and re-visit the center for further follow-ups.

At 5 month of age she visited Myelin Disorders Clinic, Children’s Medical Center, Tehran, Iran for further investigations due to her clinical findings. Motor developmental regression and recurrent seizures were noted by taking her history. Microcephaly (39.5 cm, <− 1 Z score) was also observed. Neurological examinations indicated head lag and truncal hypotonia, mild to moderate degree of spasticity in her four extremities, bilateral increased knee and ankle deep tendon reflexes with low frequent ankle clonus. Gross Motor Function Classification System (GMFCS) score was 4/5.

### Genetic study

For this purpose, Blood samples were taken from proband and her parents. DNA extraction was performed using salting out method. Screening of all the coding regions and exon-intron boundaries of *RMND1* and *RNASET2* genes were conducted by Sanger sequencing using the BigDye method by sequencing analyzer of ABI 3500XL model (PE Applied BioSystems, Massachusetts, USA). MutationTaster as an online software tools was applied for assessing the disease-causing potential of variants. The mutations were named using *RNASET2* gene NCBI Reference Sequencing NG_016280.1, NM_003730.4.

## Results

### Brain imaging

**Fig. 1:**
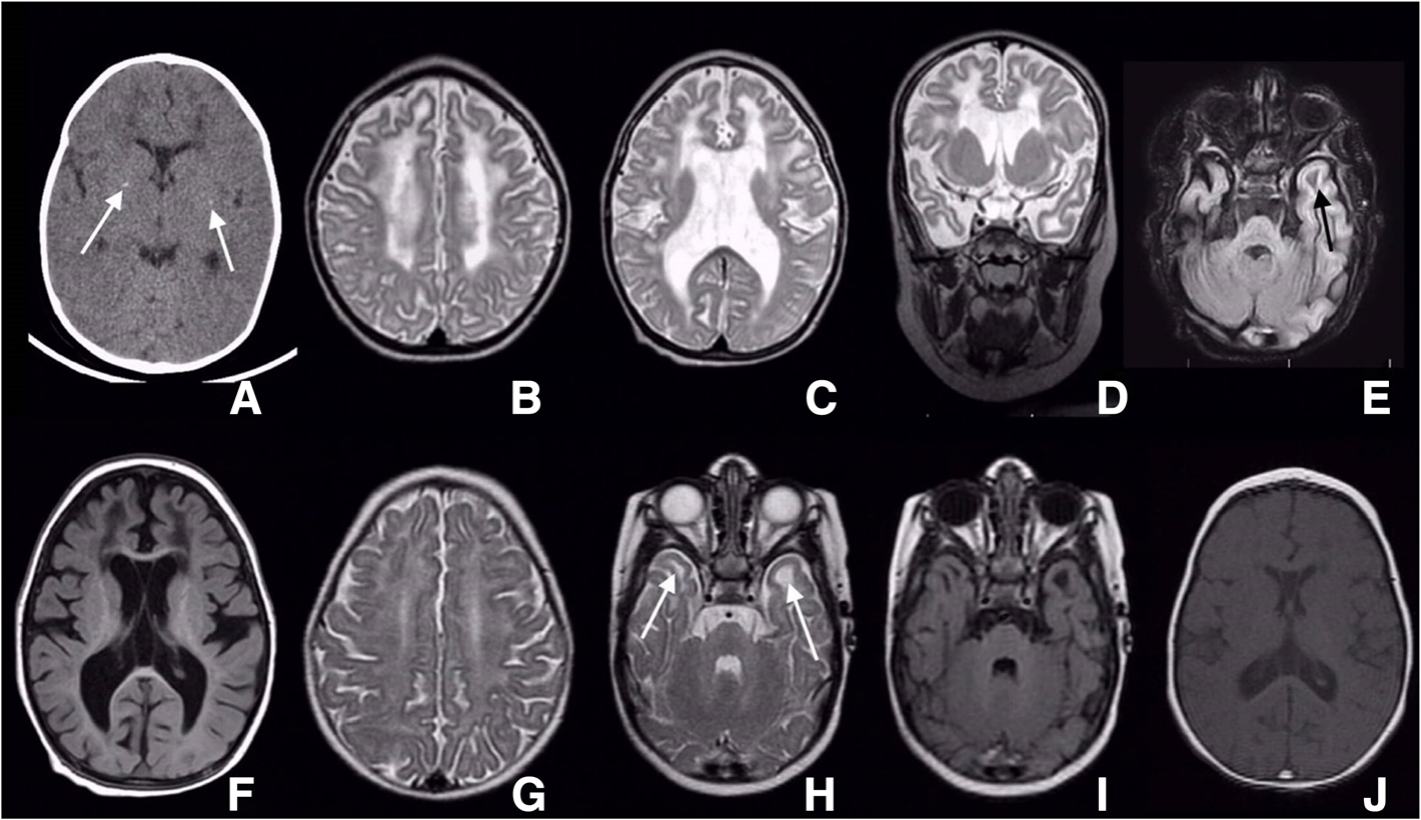
Brain CT-scan at the level of basal ganglia reveals bilateral mildly low attenuated frontal deep white matter with tiny calcified foci in the basal ganglia, most probably along lenticulostriate branches. Persistent cavum septum pellucidum is seen (white arrow) (**a**). Brain MRI, axial and coronal SE T2-WI and axial FLAIR sequence, illustrates diffuse brain volume loss with secondary ex vacuo type ventriculomegaly. Diffuse abnormal white matter signal, high in T2 WI an FLAIR which is more marked in frontal and temporal lobes is noticeable (**b**–**d**). Left anterior temporal lobe and bilateral frontal lobes cystic changes at the white matter could be seen furthermore in FLAIR sequence (black arrow) (**e**, **f**). Brain MRI, axial SE T2-WI at the level of centrum semiovale shows diffuse abnormal high signal white matter (**g**). Brain MRI, axial SE T2-WI and FLAIR sequence, at the level of lower temporal lobes reveals diffuse abnormal high signal white matter in T2-WI with bilateral subcortical anterior temporal cysts confirmed in FLAIR sequence (**h**, **i**). Brain MRI, axial SE T1-WI at the level of septum pellucidum demonstrates bilateral abnormal faint low attenuated deep white matter at frontal lobes. Persistent cavum septum pellucidum and vergae are additional findings (**j**)

Brain computed tomography (CT) scan without contrast at the first visit at 5 months of age revealed bilateral periventricular hypodence white matter lesions with fine bilateral basal ganglia calcification (Fig. 1a). One month later, brain MRI was performed and revealed a hypomyelinating pattern in deep centrum semiovale white matter, bilateral frontal white matter demyelination, cystic changes in anterior temporal area and involvement of splenium of corpus callosum (Fig. 1b–f).

### Lab tests and molecular study

**Fig. 2:**
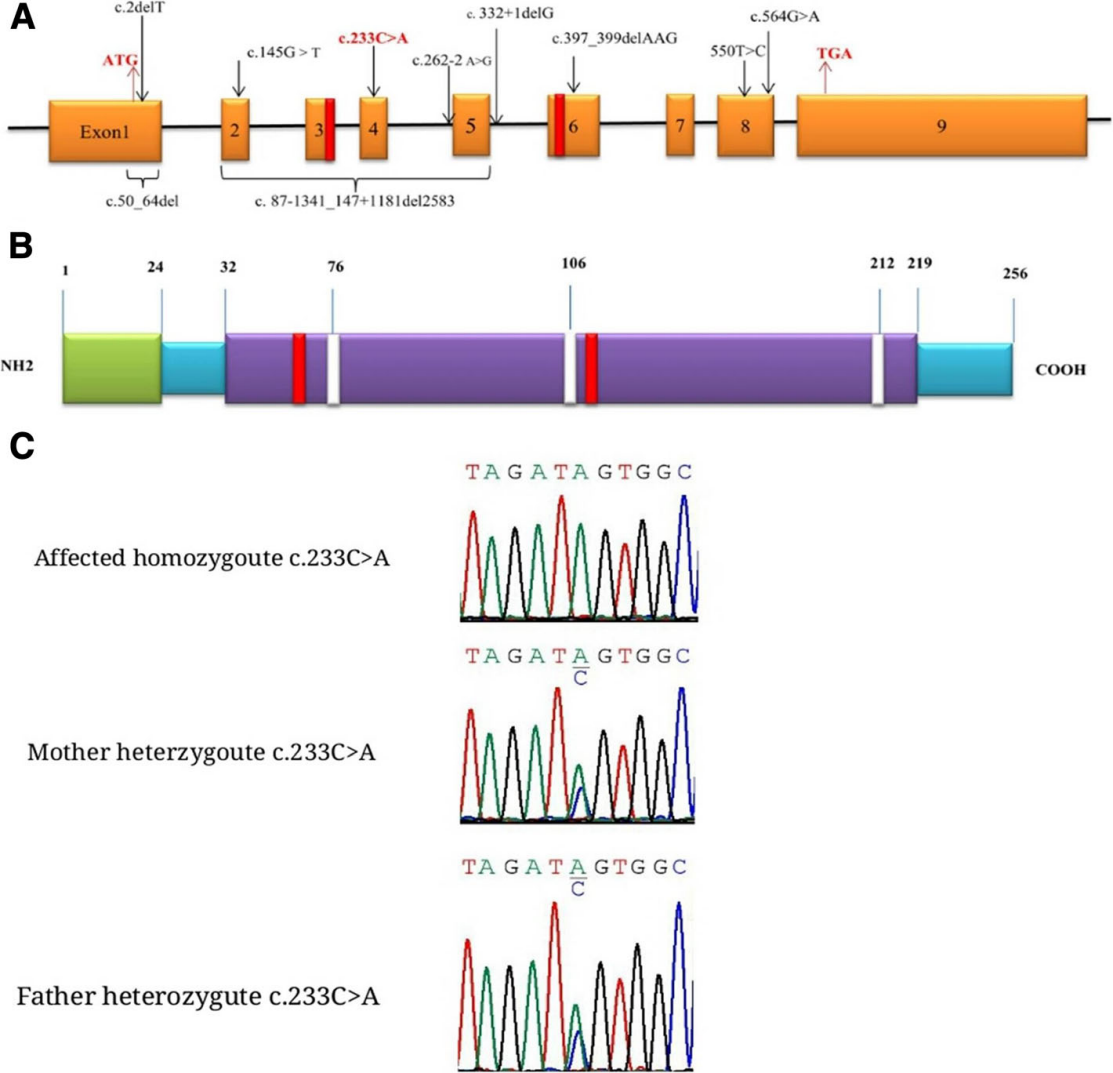
Schematic presentation of *RNASET2* gene and location of the reported mutations.1–9: exons; Red boxes: catalytic active sites (CAS I and CAS II) (**a**). Schematic presentation of RNASET2 protein. Green box: signal peptide; purple boxes: functional domains; white boxes: N- glycosilation site (**b**). Sequence chromatogram showing homozygote and heterozygote state of c.233C > A mutation in *RNASET2* (NM_ 003730.4) in the parents and affected girl

Serum and urine polymerase chain reaction (PCR) analyses for CMV infection were negative. Basic metabolic tests including serum ammonia, lactate, serum amino acids chromatography by High Performance Liquid Chromatography (HPLC) method and metabolic screen test (MS/MS) were also normal. Ophthalmic examination showed no abnormal condition. Due to the brain MRI pattern of our participant and motor regression, genetic study for leukoencephalopathies associated with anterior temporal cyst changes was considered. Molecular study revealed a homozygous variant of c.233C > A; p.Ser78Ter in *RNASET2* gene which confirmed the RNASET2-deficient leukoencephalopathy in our patient. Parents were also shown to be heterozygous for this variant (Fig. 2). Palliative therapy including rehabilitation programs were considered for the patient and anti-seizure medications were continued.

### Follow-up visits

In the follow-up visit at the age of 13 months, improvement in motor milestones regarding neck holding and rolling over were noticed but no significant change in her spasticity was observed. GMFCS grade was 3/5 and she had social smile. At the age of 19 months she had babbling, started to creep on her stomach but was unable to sit. Due to bulbar dysfunction, nasogastric tube was inserted. No change in her GMFCS score was seen. At the age of 22 months in addition to previous abilities, she gained some improvement in social interactions; she was able to identify her parents and vowel toys could attract her attention. Auditory brainstem response (ABR) test was normal. Seizures were controlled with phenobarbital and levtiracetam. Diazepam was discontinued due to increased salivary secretion. Physical examination demonstrated microcephaly with head circumference of 42.5 cm (Z score < − 2), axial hypotonia, spastic quadriparesia, bilateral esotropia and contracture of both ankle joints. GMFCS was determined to be 3–4/5. Brain MRI was repeated at the age of 22 months and revealed several signal abnormalities in centrum semiovale deep white matter in favor of hypomyelinating pattern. In addition, bilateral frontal demyelinating signals, bilateral anterior temporal cysts and black dots in putamen area indicative for calcification were noticed (Fig. 1g–j). In the last follow-up visit at the age of 31 months, GMFCS score was 4/5 and deep tendon reflexes for both knees were + 4.

## Discussion

RNASET2-deficient leukoencephalopathy is a rare neurodegenerative disorder due to bi-allelic mutations in *RNASET2* gene which is mapped on chromosome 6 [2, 7, 8]. RNASET2 is a member of the Rh/T2/S family of RNases enzymes and have various roles in different species [4]; it is responsible for transfer-RNA (tRNA) turnover in yeast and rRNA degradation in zebra fish [1]. In addition, some studies have revealed anti-angiogenic and anti-tumorigenic effects of RNASET2 which is independent from its ribonuclease activity [3, 10, 11]. In human, RNASET2 defect causes mainly neurological manifestations which mostly appear during the first year of life [7].

The diagnosis of the present case was confirmed by molecular assay at an early age and she has been under follow-up for more than 2 years. Recurrent seizures, psychomotor delay and regression, microcephaly, spasticity, and truncal hypotinia were the main clinical findings. Genetic study showed a homozygous variant of c.233C > A in exon 4 of *RNASET2* gene consistent with RNASET2-deficient leukoencephalopathy in our patient. The c.233C > A was found to be heterozygous in her parents. This mutation leads to amino acid change of p.Ser78Ter which has not been reported in studies. In-silico analysis tools predict it as a disease-causing variant.

We identified twelve genetically proven cases of RNASET2-deficient leukoencephalopathy from three previous studies [6–8] (Table 1). Former studies also described overlapping clinical and radiologic features of RNASET2-deficient leukoencephalopathy with CMV infection [6] and AGS [8]. Overall, the clinical and brain imaging data in our case were compatible with findings of other reported RNASET2-deficient leukoencephalopathy individuals.

**Table 1:** Clinical, molecular and MRI characteristics of genetically proven cases of RNase T2-deficient leukoencephalopathy and present case

Cases	Study	Nucleotide position change	Zygosity	Clinical presentations	MRI/ CT Findings
1–7	Henneke et al-2009	1,2: 550 T > C 3,4: 87-1341_147 + 1181del2583 5: 262–2 A > G 6: 332 + 1delG 7: 50_64del 567G4A	Cases 1–6: Homozygote Case 7: Compound heterozygote	-Static encephalopathy -Normo/microcephaly -Psychomotor delay	Brain MRI: - Multifocal bilateral white matter lesions - Anterior temporal subcortical cysts -Focal temporal horn enlargement - Scattered intra-cranial calcifications - Gyral abnormalities
8	Davide Tonduti et al-2016	c.550 T > C/p.Cys184Arg	Homozygote	-Age of onset: 11 months old -Generalized epileptic seizures -Psychomotor retardation -Bilateral spasticity -Truncal hypotonia -Poor social contact -Optic atrophy, and nystagmus -Microcephaly	Brain CT scans: (at 8 and 11 months of age): -Cerebral and cerebellar atrophy -Calcifications in the globus pallidus and cerebellum -White matter hypodensities in brain CT brain
9	Davide Tonduti et al-2016	c.550 T > C/p.Cys184Arg	Homozygote	-Age of onset: 15 months old -Psychomotor delay, Developmental progress at 5 years of age, Attending a school with learning difficulties -Last follow-up (20 years): -Normally grown male -Speak simple sentences -Mobile without aids -Visual and auditory function: normal -No seizures.	Brain CT: -Basal ganglia and cerebellum calcifications - Mild cerebral and cerebellar atrophy - Multifocal symmetrical subcortical white matter signal changes -Temporal and frontal lobes small cysts Brain MRI (at age 20 years): - Minimal cerebellar and cerebral atrophy -multifocal, symmetric T2 hyperintensities in the periventricular and subcortical white matter -Small cysts in the temporal lobes with larger cystic areas in both frontal lobes Last brain CT: no calcifications.
10	Davide Tonduti et al-2016	paternally: c.397_399delAAG/p.Lys133del maternally: c.145G > T/p.Glu49	Compound heterozygote	-Age of onset: 3 months old Microcephaly, pyramidal and extrapyramidal impairment, startle reaction, well social interaction, developmental progress in terms of head control at age 23 months, able to crawl and babbling 7 months later -Last follow-up (3 years of age): -Stable neurologic condition, severe spastic dystonic tetraplegia	Brain MRI (At 3 months of age): -Significant multifocal white matter abnormalities in periventricular and deep areas particularly in frontotemporal region -Follow up (15 months): white matter swelling decreased but the same white matter abnormalities Brain CT (13 months): extensive corticosubcortical cerebellar calcifications -Punctuate calcifications in the basal ganglia
11	Davide Tonduti et al-2016	c.2delT/p.Met1?	Homozygote	-Age of onset: 6 weeks old with unexplained fever, marked irritability, axial hypotonia and limb hypertonia, disappearance of systemic features with time -Developmental progress: ability to sit (2 years old), standing with support (4 years old), and walking (6 years old) -Last follow-up (11 years old): -Stable motor phenotype -Cognitive evaluation performed (between 3 and 10 years old): increasing difficulties -Autoimmune thyroiditis -Positive antinuclear antibodies -Mildly positive anti-dsDNA antibodies	Initial and follow-up MRI: - Mainly frontotemporal multifocal white matter lesions - Subcortical temporal and frontal cysts
12	Davide Tonduti et al-2016	c.2delT/p.Met1?	Homozygote	-Age of onset: 6 months old -Horizontal nystagmus -Mild psychomotor delay -Spastic paraparesis -Developmental progress: -Walk independently (spastic gait) for short distances -Developmental IQ (21 months old): low for motor functions but normal for language and sociability	Brain MRI(2.5 months of age): - Anterior predominance multifocal hyperintensity on T2 and hypointensity on T1 weighted imaging, - Posterior periventricular and temporal subcortical white matter lesions without cysts - Mild ventricular enlargement
13	Present case	c.233C > A	Homozygote	Age of onset: 3.5 month old -Afebrile tonic spasm seizures - Regression in motor milestones -Unable to neck holding - Microcephaly -Spasticity of four extremities, -Hyperreflexia and low frequent clonus in ankle joints -Head lag, truncal hypotonia -Sluggish eye fix and follow Developmental progress (13 and 19 month old): -Improvement in neck holding, sound production, and social interaction but bulbar dysfunction and feeding was with NG-Tube Last follow-up (22 months old) -Previous abilities - Seizures were controlled - Persistent microcephaly, mild axial hypotonia, spastic quadriparesia, bilateral esotropia, and mild ankle joints contracture	Brain CT (5 month old): -Bilateral periventricular hypodencity in deep white matter -Bilateral basal ganglia calcification Brain MRI (5 months old): - Deep white matter hypomyelination - Bilateral frontal white matter demyelination - Bilateral anterior temporal cyst - Splenium of corpus callosum involvement Brain MRI (22 months old): -Deep white matter signal abnormalities (Hypomyelination) -Bilateral frontal white matter demyelination (Demyelination) - Bilateral anterior temporal cysts -Bilateral putamen black dot

All mutations identified in former studies in the *RNASET2* gene did not localize to any hot spot region of this gene and they were spreading throughout all its exons. Almost all types of mutations including missense, nonsense, splice-site and deletions were reported and all of them were homozygote due to consanguineous marriages except for two cases with compound heterozygote mutations [7, 8]. These mutations possibly impaired the function of RNASET2 by disrupting disulfide bonds, exon skipping leading to frameshifts, and affecting the catalytic active sites of the protein [7]. The c.233C > A in our case was a nonsense mutation which produced a truncated and non-functional RNASET2 protein. It was postulated that this novel nonsense variant is responsible for psychomotor developmental delay and abnormal white matter signal alterations in our participant (Fig. 2).

## Conclusion

RNASET2-deficiency is a possible diagnosis in an infant presented with a static leukoencephalopathy and white matter involvement without megalencephaly; specially in an offspring of consanguineous relations. However, more common causes including CMV infection and AGS should be ruled out via clinical history, laboratory data and appropriate genetic tests.

## Data Availability

Data are available by request.

## Abbreviations

ABR: Auditory brainstem response
AGS: Aicardi-Goutieres syndrome
CMV: Cytomegalovirus
CT: Computed Tomography
GMFCS: Gross Motor Function Classification System score
HPLC: High Performance Liquid Chromatography
MRI: Magnetic Resonance Imaging
MS/MS: Metabolic screen
PCR: Polymerase chain reaction
